# Associations Between Community Social Vulnerability or Socioeconomic Deprivation and Respiratory Virus Infection in Decedents in a Large, Urban Medical Examiner’s Office

**DOI:** 10.1007/s40615-025-02482-x

**Published:** 2025-05-20

**Authors:** Xinnuo Zhao, Carl J. Schmidt, Jon Zelner, Emily T. Martin, Marisa C. Eisenberg, Paul R. Lephart, Andrea Jaworski, Allecia M. Wilson, Andrew F. Brouwer

**Affiliations:** 1 Department of Epidemiology, University of Michigan, 1415 Washington Heights, Ann Arbor, MI 48130 USA; 2 Department of Pathology, University of Michigan, Ann Arbor, MI USA; 3 Department of Pathology, Wayne State University, Detroit, MI USA

**Keywords:** Decedent, Respiratory virus, Social vulnerability index, Area deprivation index, Health disparity

## Abstract

**Background:**

Current respiratory virus surveillance relies primarily on medically attended, symptomatic cases, which may distort the true patterns of respiratory virus infection. Estimating the underlying respiratory viral infection patterns, regardless of the symptomatic status, may help us to better intervene and address issues of racial and ethnic health equity. We tested decedents from the Wayne County Medical Examiner’s Office (Wayne & Monroe Counties, Michigan including Detroit) regardless of the cause of death.

**Methods:**

Nasopharyngeal samples were collected from decedents at the Wayne County Medical Examiner’s Office between October 2020 and September 2022 and tested for a panel of respiratory viruses. We identified 3430 decedents with catchment addresses, which we linked to the social vulnerability index (SVI) and area deprivation index (ADI) through US Census tract/block. We evaluated non-linear associations between each of adenovirus (AdV), SARS-CoV-2, parainfluenza virus 2 (PIV2), rhinovirus (RV), and respiratory syncytial virus (RSV) prevalence and ADI, SVI, and SVI subthemes using splines in log-binomial regression models.

**Results:**

There were few statistically significant associations observed between overall SVI or ADI and respiratory virus infection prevalence. However, RV was significantly associated with ADI, SVI, and most SVI subthemes. The SVI Minority Status and Language (MSL) subtheme was statistically significantly associated with the prevalence of AdV and RV (*p* < 0.05) and associations with SARS-CoV-2 and RSV approached significance (*p* < 0.10).

**Conclusions:**

Our results suggest that the MSL subtheme of SVI may be the most informative community-level predictor of respiratory virus infections and could be used to prioritize health-equity-focused distribution of public health resources.

**Supplementary Information:**

The online version contains supplementary material available at 10.1007/s40615-025-02482-x.

## Introduction

Lower respiratory infections (including pneumonia and bronchiolitis) are the leading cause of death among children under 5 years old globally and the 6th leading cause of all-ages mortality [1]. In 2016, there were 0.6 million deaths due to lower respiratory infection in children under 5, 1.1 million in adults over 70, and 2.4 million overall [2]. Pneumonia alone is responsible for more deaths than many higher profile diseases combined, including malaria, Zika, Ebola, HIV, and tuberculosis [3]. In the USA, acute respiratory infections (ARI) make up more than 50% of children’s infectious-disease-related emergency room visits (41% upper respiratory infection, 14% lower respiratory infection) ^4^. Approximately 500 per 100,000 children with infectious-disease-related emergency visits are hospitalized, and 40% of these are related to lower respiratory infections (60% among infants) [4]. ARIs are caused by a wide variety of respiratory viruses, including rhinovirus (RV), respiratory syncytial virus (RSV), influenza virus (IV), parainfluenza virus (PIV), human metapneumovirus (hMPV), adenovirus (AdV), and coronavirus (CoV) [5]. The Pneumonia Etiology Research for Child Health (PERCH) Study estimated that about 60% of pneumonia has a viral etiology [6]. Altogether, viral respiratory infection is a major cause of morbidity and mortality both in the USA and globally.

One difficulty in studying respiratory virus infections is that we do not determine the etiology of most instances of respiratory disease, and, even when samples are tested and submitted to active case surveillance systems, the data are biased by treating-seeking behavior and patterns of asymptomatic infection. Therefore, it has been difficult to fully determine and understand the disparities, particularly racial and ethnic disparities, in respiratory virus infection [7–9]. The COVID-19 pandemic brought racial and ethnic disparities into high relief, as Black and Hispanic individuals in the USA were at substantially the higher risk of SARS-CoV-2 infection and hospitalization than white individuals, especially in the first wave of infections [10–12]. Beyond SARS-CoV-2, racial and ethnic disparities have been reported in respiratory illness and hospitalizations, both all-cause and for some specific infections such as influenza and respiratory syncytial virus [7, 13, 14].

Racial and ethnic disparities are influenced by a complex network of interrelated socioeconomic factors, such as poverty, access to transportation, crowded housing, education access, employment and housing quality [15, 16]. Accordingly, social vulnerability indices have been developed to attempt to quantify the risk to—and resilience of—communities in the face of external stresses [17]. In the USA, two such indices include the social vulnerability index (SVI) [18], developed by the Centers for Disease Control and Prevention and the Agency for Toxic Substances and Disease Registry, and the area deprivation index (ADI), which has been used by several federal agencies [19]. SVI assesses multiple community-level social factors, including socioeconomic status, household characteristics, racial and ethnic minority status, and housing type and transportation, and is defined at the census tract level. SVI has been widely used in allocating emergency funding and estimating supplies needed for low-SVI communities in an event of disaster [18]. ADI ranks census blocks by metrics of socioeconomic advantage or disadvantage including income, education, employment, and housing quality [19]. Neither SVI nor ADI directly accounts for individual-level risk or protective factors and behaviors, but they do provide context for the community in which individuals are located, as well as possible avenues for pursuing equitable distribution of public health resources.

In this exploratory analysis, we estimated non-linear associations between each of the five most prevalent respiratory virus infections in decedents in Wayne and Monroe counties, Michigan over a 2-year period (SARS-CoV-2, RV, AdV, RSV, and PIV2, with prevalence ranging from 1 to 16%) and the SVI and ADI values of the census area of the decedents’ residences. By sampling decedents regardless of cause of death, we can look at patterns of respiratory virus infection unbiased by asymptomatic infection, care-seeking behavior, or healthcare access. Specifically, we are interested in determining whether there are patterns in respiratory virus infection by the relative social vulnerability or socioeconomic deprivation of decedents’ communities and the extent to which such patterns may explain racial and ethnic disparities in infection. Our work will contribute to a better understanding of the patterns and drivers of disparities in respiratory virus infection, not just outcomes, and thus will ultimately contribute to efforts to reduce those disparities.

## Methods

### Population and Sample Collection

The forensic pathologists in a Medical Examiner’s Office (MEO) are charged with determining the manner of death in events of sudden, violent, suspicious, or unexpected deaths. Decedents are not a representative sample of the population: men, Black individuals, and adults ages 18–64, are overrepresented among decedents compared to the catchment population of the Wayne County Medical Examiner’s Office (WCMEO) [9, 12]. In addition to the selection bias caused by differential death rates across demographics, it is possible that respiratory virus infection itself affects one’s likelihood of dying and entering the MEO (although deaths attributed to respiratory infection are rare, we previously reported differences in respiratory virus prevalence by manner of death more generally [9].) Additionally, by the nature of the MEO, decedents are exposed to violence at a higher rate than the general population, and the conditions associated with higher likelihood of violence could also be associated with a higher risk of respiratory virus infection. Accordingly, our results in this analysis should be seen as representing the population of decedents, rather than the catchment population more broadly.

As previously reported, the WCMEO, which services Wayne and Monroe Counties in Michigan, collected up to 10 nasopharyngeal daily between October 2020 and September 2022 from decedents [9]. A total of 4121 samples were collected during this period. Decedents with significant head trauma, anterior lividity, or signs of decomposition were excluded from testing. We characterized decedent demographic variables including age, sex, race/ethnicity (Black, White, Hispanic, or Other/Unknown), manner of death (natural, accident (including drug-related deaths), homicide/suicide, or pending/indeterminate). The address of residence was used to link participants to geographic socioeconomic and social vulnerability indicators, as discussed below. MEOs are exempt from the Health Insurance Portability and Accountability Act (HIPAA), and MEO records are publicly available by Freedom of Information Act (FOIA) request in Michigan. This study was determined to be not regulated as human subjects research by the University of Michigan Institutional Review Board (HUM00239558) as decedents are not human subjects.

### Respiratory Virus Testing

Decedent samples were tested by BioFire FilmArray Respiratory PCR Panel 2.1 (BioFire RP2.1, BioFire Diagnostics, bioMérieux, Marcy I’Etoile; Etoile, France) for the following 16 respiratory viruses: adenovirus (AdV); SARS-CoV-2; seasonal coronaviruses (CoV) 229E, NL63, OC43, and HKU1; human metapneumovirus (hMPV); influenza A virus (IAV) subtypes H3 and H1; influenza B virus (IBV); parainfluenza virus (PIV) 1, 2, 3, and 4; rhinovirus/enterovirus (RV); and respiratory syncytial virus (RSV). The 8 decedents with invalid results were removed from the sample, leaving 4113. Prevalence of each virus and association with age, sex, race and ethnicity, and manner of death were previously reported [9]. Only viruses with at least 1% prevalence across all samples were included in this analysis to ensure adequate sample size for statistical inference: these viruses were SARS-CoV-2 (15.7%), RV (11.2%), AdV (4.9%), PIV2 (1.3%), and RSV (1.2%).

### Community Characteristics: Social Vulnerability and Socioeconomic Deprivation

Decedent data were cleaned by filtering out invalid addresses, including addresses that were not residences, missing addresses, or addresses outside the catchment area (i.e., outside of Wayne and Monroe counties Michigan); 683 observations were excluded. The remaining 3430 decedents, who all had complete demographic information, were linked to the SVI and ADI by matching US census tract and block federal information processing standard codes (FIPS) through the US Census Geocoder. SVI measures the potential negative effects of external stress to human health on communities and includes four subthemes: Socioeconomic Status (SES), Household Composition and Disability (HCD), Minority Status and Language (MSL), and Housing Type and Transportation (HTT). SVI ranges from 0 to 1, with higher SVI values associated with greater social vulnerability. ADI is a measure of the level of socioeconomic deprivation in a community and has a scale of 0 to 100, with higher ADI values associated with higher levels of socioeconomic deprivation. For descriptive statistics, the community characteristics were discretized into 5 categories of equal width.

### Statistical Analysis

We calculated the prevalence of each infection by each individual demographic characteristic and across levels of the discretized community characteristics. Descriptive associations between infection prevalence and each of the individual and community covariates were assessed by the chi-squared test or, where any cell size was < 5, Fisher’s exact test for categorical characteristics with more than 2 levels or by test of proportions for characteristics with 2 levels.

When estimating non-linear associations between a continuous predictor and an outcomes of interest, categorizing the continuous predictor creates several statistical and inference problems, including implausible discontinuities across categories [20]. An alternative approach is to model the non-linear association using splines, which are smooth piecewise polynomials [20]. We estimated non-linear associations between continuous ADI, SVI, and the SVI themes, each modeled using cubic B-splines [20] with 3 degrees of freedom (see below), and infection of each of AdV, SARS-CoV-2, PIV2, RV, and RSV in log-binomial regression models. The results of these models are smooth estimates of infection prevalence of each virus as a function of each continuous predictor. We used likelihood ratio tests (LRT) to determine if there was an association between the predictors and the outcomes (i.e., whether the spline regressions fit statistically significantly better than a model with an intercept only). To contextualize the spline regression fits, we also calculated infection prevalence for each virus on deciles of each community characteristic.

We chose 3 degrees of freedom for the spline models based on a sensitivity analysis (Supplemental Table S1), calculating the Bayesian Information Criterion (BIC) to balance model fit and parsimony across all models, comparing to 3–5 degrees of freedom. We found 3 degrees of freedom, which is the lowest allowable for cubic B-splines, had the best balance of model fit and parsimony (i.e., lowest BIC) in all models.

Additionally, we evaluated associations between infection with each respiratory virus with decedent sociodemographic characteristics, manner of death, and quintiles of ADI, SVI, and SVI subcategories in univariable and multivariable Poisson regression models with robust error variance, estimating prevalence ratios (Poisson regression with robust error variance is an alternative when log-binomial regression is non-convergent) [21]. The multivariable models accounted for sociodemographic characteristics and manner of death as well as SVI MSL, based on the crude associations. Statistical analysis was done by using R (v 4.2.1).

In this exploratory study, we highlight results both that are statistically significant at the level of significance *α* = 0.05 and at the level of significance *α* = 0.10. Because this study is hypothesis generating, not hypothesis testing, we do not adjust for multiple comparisons [22].

## Results

The individual and community characteristics of the decedents are reported in Table 1. As previously reported, decedents are more likely to be male (68% of this sample; 51% of catchment), Black individuals (53% of sample; 35% of catchment), and adults aged 18–64 years (83% of sample; 60% of catchment) [9, 12]. Approximately half (49%) of decedents came from communities with the highest category of social vulnerability (SVI > 0.8), and two-thirds (68%) came from communities with the category of greatest socioeconomic deprivation (ADI > 80).

**Table 1:** Prevalence of each respiratory virus among decedents in the Wayne County Medical Examiner’s Office (2020–2022). The *p*-values are for differences in prevalence by characteristic as calculated by chi-square test or Fisher’s exact test when any count < 5 for categorical variables with more than 2 levels (age, race/ethnicity, manner of death, social vulnerability index (SVI), SVI subthemes, and area deprivation index (ADI)) or test of proportions for categorical variables with 2 levels (sex). Virus abbreviations: adenovirus (AdV), SARS-CoV-2, parainfluenza virus 2 (PIV2), rhinovirus (RV), and respiratory syncytial virus (RSV)

Characteristic	Sample	Infection prevalence				
		AdV	SARS-CoV-2	PIV-2	RV	RSV
	*N* (%) of sample	*n* (%) positive	*n* (%) positive	*n* (%) positive	*n* (%) positive	*n* (%) positive
	3430 (100%)	161 (4.7%)	552 (16%)	43 (1.3%)	375 (11%)	41 (1.2%)
Age (years)		***p* < 0.001**	*p* = 0.227	***p* < 0.001**	***p* < 0.001**	***p* = 0.001**
0–4	138 (4.0%)	12 (8.7%)	27 (20%)	4 (2.9%)	71 (51%)	6 (4.3%)
5–17	50 (1.5%)	11 (22%)	11 (22%)	5 (10%)	18 (36%)	2 (4.0%)
18–39	1081 (32%)	79 (7.3%)	184 (17%)	20 (1.9%)	160 (15%)	16 (1.5%)
40–64	1736 (51%)	50 (2.9%)	273 (16%)	14 (0.8%)	112 (6.5%)	16 (0.9%)
65 +	425 (12%)	9 (2.1%)	57 (13%)	0	14 (3.3%)	1 (0.2%)
Sex		***p* = 0.008**	*p* = 0.711	***p* = 0.005**	***p* = 0.015**	*p* = 0.088
Female	1082 (32%)	66 (6.1%)	178 (16%)	22 (2.0%)	139 (13%)	18 (1.7%)
Male	2345 (68%)	95 (4.0%)	374 (16%)	21 (0.9%)	236 (10%)	23 (1.0%)
Race/ethnicity		***p* = 0.008**	***p* = 0.025**	***p* < 0.001**	***p* < 0.001**	***p* = 0.004**
Black	1814 (53%)	104 (5.7%)	315 (17%)	33 (1.8%)	237 (13%)	28 (1.5%)
Hispanic	94 (2.7%)	6 (6.4%)	20 (21%)	1 (1.1%)	16 (17%)	3 (3.2%)
White	1444 (42%)	49 (3.4%)	202 (14%)	6 (0.4%)	112 (7.8%)	8 (0.6%)
Other/unknown	78 (2.3%)	2 (2.5%)	15 (19%)	3 (3.8%)	3 (7.7%)	2 (2.5%)
Manner of death		***p* = < 0.001**	***p* = < 0.001**	*p* = 0.286	***p* < 0.001**	*p* = 0.680
Natural	1328 (39%)	44 (3.3%)	270 (20%)	13 (1.0%)	91 (6.9%)	14 (1.1%)
Accident	1565 (46%)	76 (4.9%)	205 (13%)	20 (1.3%)	201 (13%)	20 (1.3%)
Violent	352 (10%)	28 (8.0%)	52 (15%)	8 (2.3%)	49 (14%)	6 (1.7%)
Indeterminate/pending	185 (5.4%)	13 (7.0%)	25 (14%)	2 (1.1%)	34 (18%)	1 (0.5%)
SVI: overall		*p* = 0.556	*p* = 0.766	*p* = 0.477	***p* < 0.001**	*p* = 0.199
0.0–0.20	201 (5.9%)	10 (5.0%)	26 (13%)	3 (1.5%)	46 (6.7%)	0 (0.0%)
0.21–0.40	360 (10%)	17 (4.7%)	56 (16%)	4 (1.1%)	85 (12%)	7 (1.9%)
0.41–0.60	328 (9.6%)	10 (3.0%)	53 (16%)	4 (1.2%)	68 (9.9%)	3 (0.9%)
0.61–0.80	850 (25%)	37 (4.4%)	137 (16%)	6 (0.7%)	95 (14%)	7 (0.8%)
0.81–1.00	1691 (49%)	87 (5.1%)	280 (17%)	26 (1.5%)	81 (12%)	24 (1.4%)
SVI: socioeconomic status		*p* = 0.096	*p* = 0.491	*p* = 0.444	***p* < 0.001**	*p* = 0.603
0.0–0.20	209 (6.1%)	14 (6.7%)	28 (13%)	3 (1.4%)	44 (6.4%)	4 (1.9%)
0.21–0.40	255 (7.4%)	10 (3.9%)	43 (17%)	4 (1.6%)	71 (10%)	1 (0.4%)
0.41–0.60	365 (11%)	9 (2.5%)	50 (14%)	4 (1.1%)	71 (10%)	3 (0.8%)
0.61–0.80	571 (17%)	23 (4.0%)	98 (17%)	3 (0.5%)	91 (13%)	7 (1.2%)
0.81–1.00	2030 (59%)	105 (5.2%)	333 (16%)	29 (1.4%)	98 (14%)	26 (1.3%)
SVI: household composition and disability		*p* = 0.450	*p* = 0.361	*p* = 0.283	*p* = 0.287	*p* = 0.742
0.0–0.20	321 (9.4%)	16 (5.0%)	44 (14%)	6 (1.9%)	100 (9.3%)	4 (1.2%)
0.21–0.40	407 (12%)	17 (4.2%)	56 (14%)	4 (1.0%)	141 (12%)	7 (1.7%)
0.41–0.60	676 (20%)	26 (3.8%)	118 (17%)	11 (1.6%)	43 (12%)	9 (1.3%)
0.61–0.80	888 (26%)	51 (5.7%)	150 (17%)	6 (0.7%)	39 10%)	8 (0.9%)
0.81–1.00	1138 (33%)	51 (4.5%)	184 (16%)	16 (1.4%)	52 (12%)	13 (1.1%)
SVI: minority status and language		***p* = 0.025**	*p* = 0.082	*p* = 0.534	***p* < 0.001**	*p* = 0.352
0.0–0.20	116 (3.4%)	5 (4.3%)	12 (10%)	0 (0.0%)	33 (5.8%)	0 (0.0%)
0.21–0.40	209 (6.1%)	9 (4.3%)	27 (13%)	2 (1.0%)	75 (11%)	0 (0.0%)
0.41–0.60	432 (13%)	15 (3.5%)	66 (15%)	3 (0.7%)	35 (8.6%)	5 (1.2%)
0.61–0.80	506 (15%)	12 (2.4%)	72 (14%)	5 (1.0%)	92 (12%)	5 (1.0%)
0.81–1.00	2167 (63%)	120 (5.5%)	375 (17%)	33 (1.5%)	140 (14%)	31 (1.4%)
SVI: housing type and transportation		*p* = 0.729	*p* = 0.829	*p* = 0.718	*p* = 0.943	*p* = 0.882
0.0–0.20	506 (15%)	24 (4.7%)	76 (15%)	7 (1.4%)	171 (11%)	4 (0.8%)
0.21–0.40	580 (17%)	27 (4.7%)	89 (15%)	6 (1.0%)	108 (11%)	6 (1.0%)
0.41–0.60	730 (21%)	29 (4.0%)	124 (17%)	7 (1.0%)	46 (11%)	10 (1.4%)
0.61–0.80	781 (23%)	43 (5.5%)	123 (16%)	9 (1.2%)	29 (12%)	10 (1.3%)
0.81–1.00	833 (24%)	38 (4.6%)	140 (17%)	14 (1.7%)	21 (10%)	11 (1.3%)
ADI		*p* = 0.400	*p* = 0.384	*p* = 0.779	***p* < 0.001**	*p* = 0.648
0–20	30 (0.9%)	1 (3.3%)	2 (6.7%)	0 (0.0%)	40 (5.8%)	1 (3.3%)
21–40	138 (4.0%)	4 (2.9%)	23 (17%)	2 (1.4%)	57 (8.3%)	1 (0.7%)
41–60	325 (9.5%)	17 (5.2%)	44 (14%)	5 (1.5%)	108 (14%)	4 (1.2%)
61–80	610 (18%)	21 (3.4%)	94 (15%)	5 (0.8%)	88 (14%)	6 (1.0%)
81–100	2312 (68%)	117 (5.1%)	386 (17%)	33 (1.3%)	81 (13%)	29 (1.3%)

The overall prevalence was highest for SARS-CoV-2 (16%), followed by RV (11%), and AdV (4.7%) and was lowest for RSV (1.3%) and PIV-2 (1.2%). There was a large difference in prevalence by age for RV, with prevalence ranging from > 50% for ages < 5 years to < 5% for ages 65 + years. Prevalence also statistically significantly varied by age for AdV, RSV, and PIV-2, with higher prevalence for those under age 18 and lower prevalence for adults, but it did not statistically significantly vary by age for SARS-CoV-2. Prevalence by race and ethnicity followed similar trends across viruses, with Black and Hispanic decedents having higher prevalence of all respiratory viruses compared to White decedents. In most cases, the prevalence of each respiratory virus did not statistically significantly vary over the categories of SVI, most of the SVI subthemes, or ADI. However, there were several notable exceptions. AdV and RV were statistically significantly associated with the SVI MSL subtheme (*p* < 0.05 for chi-squared test), and the association between SARS-CoV-2 and SVI MSL approached statistical significance (*p* < 0.10 for chi-squared test). For all viruses, the prevalence of infection was highest in the highest category of SVI MSL. Further, although the association was not significant by Fisher’s exact test for PIV-2 or RSV, there were no infections in the 116 decedents with the lowest values of MSL for either virus. Additionally, RV was significantly associated with overall SVI, the SVI SES subtheme, and ADI. No virus was statistically significantly associated with the SVI subthemes of HCD or HTT.

**Fig. 1:**
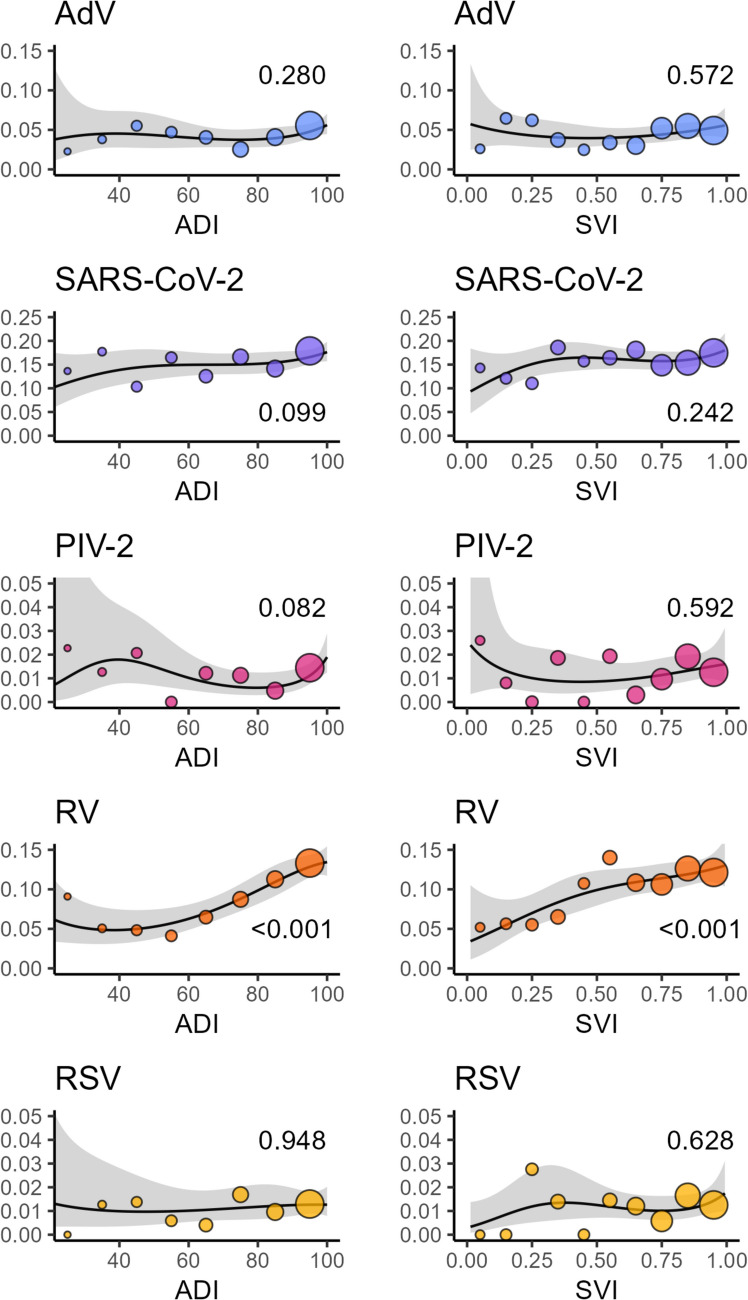
Continuous associations between respiratory virus prevalence and the area deprivation index (ADI) and social vulnerability index (SVI) among decedents in the Wayne County Medical Examiner’s Office (2020–2022). Points give the mean prevalence in the strata of 10% of the *x*-axis, with point size proportional to the number of decedents in that stratum. *p*-values are given for the likelihood ratio test comparing the nonlinear model vs a model with intercept alone. Virus abbreviations: adenovirus (AdV), SARS-CoV-2, parainfluenza virus 2 (PIV-2), rhinovirus (RV), and respiratory syncytial virus (RSV)

**Fig. 2:**
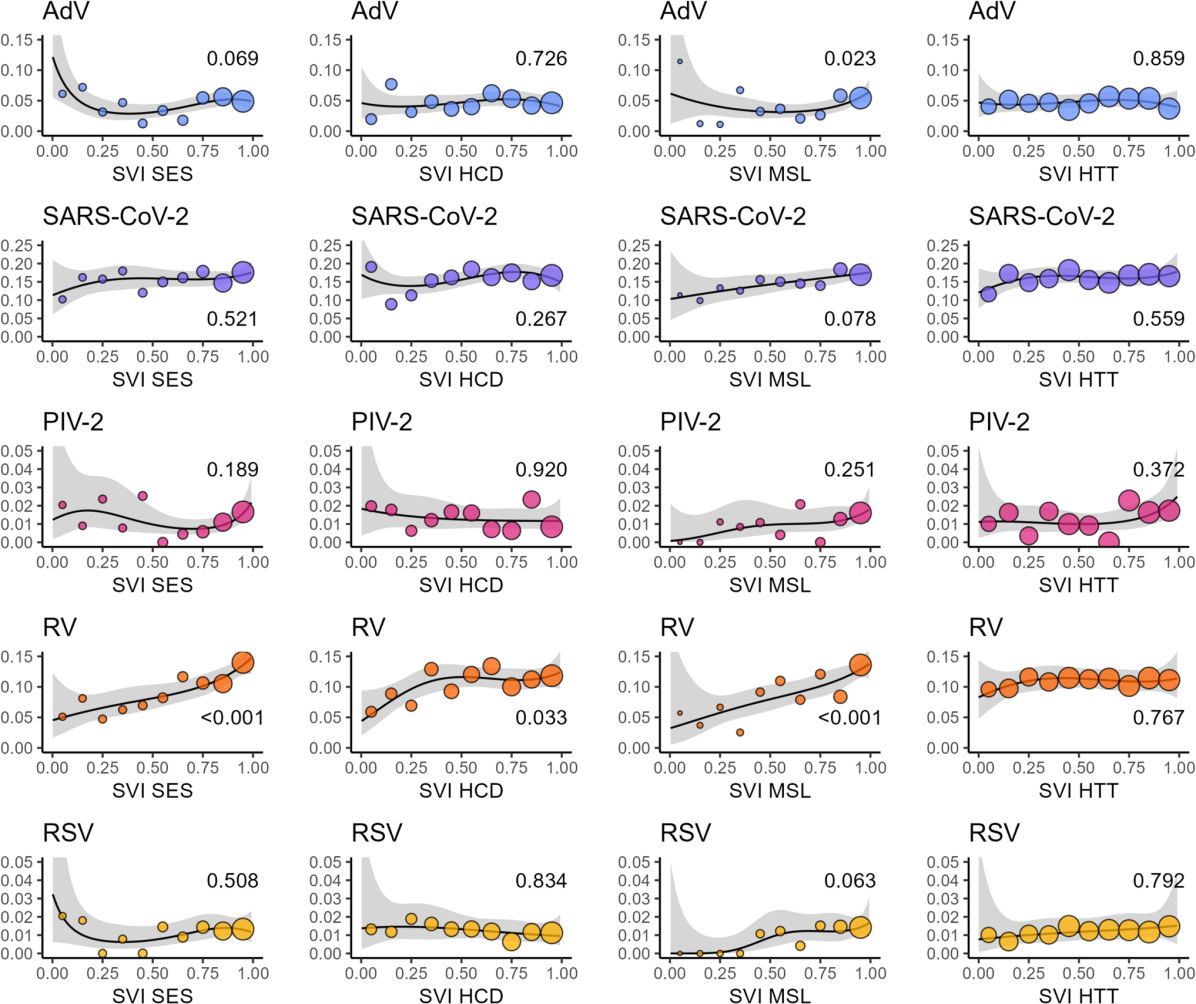
Continuous associations between respiratory virus prevalence and subthemes of the social vulnerability index (SES, socioeconomic status; HCD, household composition and disability; MSL, minority status and language; HTT, housing type and transportation) among decedents in the Wayne County Medical Examiner’s Office (2020–2022). Points give the mean prevalence in the strata of 10% of the *x*-axis, with point size proportional to the number of decedents in that stratum. *p*-values are for the likelihood ratio test comparing the nonlinear model vs a model with intercept alone. Virus abbreviations: adenovirus (AdV), SARS-CoV-2, parainfluenza virus 2 (PIV-2), rhinovirus (RV), and respiratory syncytial virus (RSV)

The non-linear associations between the prevalence of each of the respiratory viruses and each of SVI and ADI are given in Fig. 1 and associations with each SVI subtheme are given in Fig. 2. Consistent with Table 1, there was no statistically significant association between the prevalence of AdV, SARS-CoV-2, PIV-2, and RSV and the community characteristics SVI, the SVI subthemes except MSL, or ADI. For SVI MSL, most viruses showed a trend with higher prevalence with greater values of the MSL theme and lower prevalence for lower values. (AdV may be an exception to this pattern, as the spline estimate predicts a U-shaped association, although there is high uncertainty at the lower end of the MSL theme.) The association between SVI MSL and prevalence was statistically significant for AdV and RV (*p* < 0.05 for LRT), meaning that the nonlinear spline model was a better fit to the data than a constant prevalence across SVI MSL for these two viruses. Additionally, the associations for SARS-CoV-2 and RSV with MSL approached statistical significance (*p* < 0.10 for LRT). Although PIV-2 was not statistically significantly associated with MSL (*p* = 0.25 for LRT), the overall pattern is suggestive, so that the lack of association is likely driven by sample size. RV was associated with both SVI and ADI, as well as each SVI theme except for SVI HTT, with increasing prevalence with greater social vulnerability or greater socioeconomic deprivation.

We estimated the associations between SVI, its subthemes, and ADI with infection by each respiratory virus through univariable and multivariable Poisson models with robust variance (Supplemental Table S2). The associations estimated in the univariable analyses did not change substantially in the multivariable analyses, suggesting that there is little confounding of the relationships between these variables and respiratory virus prevalence. Although accounting for community MSL and individual demographic characteristics did slightly attenuate the estimated magnitude of racial and ethnic disparities, the associations were often no longer statistically significant after adjustment. Specifically, the prevalence ratio for Black vs White decedents decreased by 22% to 1.32 (95% CI, 0.86, 2.04) for AdV, by 10% to 1.11 (95% CI, 0.90, 1.37) for SARS-CoV-2, by 10% to 3.96 (95% CI, 1.33,11.8) for PIV-2, by 21% to 1.32 (95% CI, 1.00, 1.73) for RV, and by 24% to 2.13 (95% CI, 0.71, 6.36) for RSV, suggesting racial and ethnic disparities are partially but not completely confounded by the other individual demographic factors or community MSL. Similarly, many of the prevalence ratios by community MSL category did not change substantially after adjusting for the individual demographic characteristics, including race and ethnicity and age, suggesting that the effect of MSL on infection prevalence is not simply an artifact of correlations between MSL and demographics.

## Discussion

In this analysis, we estimated associations between each of five respiratory virus infections and measures of community social vulnerability or socioeconomic deprivation in an urban decedent population, with an aim of better understanding the patterns and drivers of disparities in respiratory virus infection. Consistent with our previous analysis in the larger sample (which was not subset to those with residential addresses in Wayne or Monroe counties, Michigan), there were racial and ethnic disparities in infection prevalence across the respiratory viruses [9], highlighting the disproportionate burden of respiratory virus infections among people of color. However, we found that measures of community social vulnerability or socioeconomic deprivation largely did not explain these disparities in decedents. First, SVI, its subthemes other than Minority Status and Language (MSL), and ADI of a decedent’s residential address were not statistically significantly associated with prevalence of most of the studied respiratory viruses, except for RV. Second, although the MSL subtheme of SVI was associated with prevalence of several viruses, racial and ethnic disparities were only slightly attenuated when adjusting for MSL and the other demographic factors, suggesting that community racial composition is not a strong confounder of the association between individual race and ethnicity and respiratory virus prevalence. Our results have important implications for how public health resources should be distributed to reduce health inequities.

In this analysis, we purposefully highlighted community-level predictors of infection rather than individual-level predictors, both because they provide a measure of social factors not captured by individual-level predictors and because they may offer an actionable target for public health policy. However, we found that even when community-level predictors like MSL could partially explain racial and ethnic disparities in infection, they did not explain it in full. This outcome is perhaps not unexpected: people are highly mobile and respiratory viruses highly transmissible, so exposure may not occur in one’s home community; decedent home address may be misclassified; and, as suggested by Tran et al. (2023), these community characteristics may not adequately represent the heterogeneity within communities and unique challenges faced by particular subpopulations [23]. Indeed, past work has generally found that community-level metrics can only partially explain individual outcomes. For example, Chandrasekhar et al. [14] estimated that census-tract level determinants accounted for 11% of variability in influenza hospitalization. It may also be that community-level metrics like SVI and ADI may be less informative for transmissible infectious diseases compared to chronic diseases, such as cardiovascular disease or COPD [24–26], which are the result of longer-term exposures (e.g., food deserts, lack of walkability, pollution, exposure to cigarettes) that may be better captured by one’s address. SVI has also been associated with sexually transmitted infections like HIV, gonorrhea, and syphilis [27, 28], but there is much stronger assortativity in sexual contacts (i.e., higher likely of contacts with similar demographic profiles) than respiratory transmission [29].

If not community social vulnerability and socioeconomic deprivation, what drives racial and ethnic disparities? Individual factors structurally tied to race and ethnicity—such as occupational exposures, individual health behaviors, insurance and access to care, and increased susceptibility via allostatic load [16]—may be more important predictors of infection than one’s home address. For respiratory infections and COVID-19 especially, occupational exposure has been a particular focus of recent research, as racial and ethnic minority groups may be overrepresented in certain occupations in sectors such as healthcare, grocery stores, and public transportation that may increase exposure to respiratory viruses [30, 31]. These groups may have less access to physical distancing, work-from-home options, and paid sick leave, which could contribute to higher exposure to others in the work setting. It is important to recognize that these individual factors are still being driven by broad social determinants of health; they are just not measured in one’s home address.

The MSL subtheme of SVI is based solely on the relative population in the census tract that is not non-Hispanic White and on the fraction that speak English “less than well.” [18] It may be that MSL is not reflecting the cause of racial and ethnic disparities (as potential factors like crowding or socioeconomic disadvantage are captured by other SVI themes that were not broadly associated with respiratory virus prevalence) but rather reflecting the disparities themselves, i.e., the communities have higher respiratory virus prevalence *because* there are more people of color and people of color have higher prevalence. Although this interpretation is perhaps less useful scientifically, it still does have practical implications. Indeed, there is increasing interest in finding ways to equitably distribute public health resources [32, 33]. In the context of respiratory viruses, equitable distribution of public health resources could include decisions about where to prioritize access to vaccines when supply is limited, where to locate clinics for routine testing, vaccination, or treatment, and where to focus community engagement efforts. While SVI has been used in allocation of resources and intervention for disaster preparedness, our results suggest that it may not be the optimal choice of metric to evaluate respiratory virus infection. Nevertheless, the MSL subtheme was more strongly associated with several respiratory viruses than overall SVI and ADI, suggesting that, even if it is not reflecting causes of infection, it might be more effective to use the MSL subtheme over SVI overall or ADI in distributing public health resources to reduce respiratory infection disparities in the absence of other validated measures, e.g., locating more vaccine clinics in high MSL communities. We acknowledge that while leveraging the MSL index may be scientifically the strongest choice for targeting disparities in respiratory virus infection, there may be practical and political barriers to its use.

Unlike the other respiratory viruses, the prevalence of RV *was* significantly associated with community ADI, SVI, and all of the SVI subthemes except Housing Type and Transportation (HTT). This means that factors such as community-level poverty, unemployment, housing cost burden, low education level, and low prevalence of health insurance were associated with a higher risk of RV infection in decedents. This finding is consistent with previous work that found that lower subjective socioeconomic status was associated with increased susceptibility to the common cold after exposure to rhinovirus [34]. While these indices could be capturing mechanistic risk factors for transmission—such as higher contact rates through crowding—it is unclear why the association would not hold for the other respiratory viruses, which are also primarily spread by respiratory droplets among close contacts. Rhinovirus prevalence has a strong relationship with age, and, although communities with higher social vulnerability or economic deprivation had a higher fraction of decedents ages 0–4 years, the association does not appear to be confounded by age. Previous work has suggested that poorer sleep efficiency and duration among people with lower socioeconomic status was a mediator for association between subjective socioeconomic status and common cold susceptibility [34]. It is uncertain whether or why this possible association would be unique to RV, however. More work is needed to understand these associations.

### Strengths and Limitations

The strengths of our study include our approach to estimating the true burden of respiratory viral infection by considering both symptomatic and asymptomatic cases; most studies have focused on medically attended, symptomatic cases. Examining symptomatic cases alone may not present the whole picture of the pattern of respiratory viral infection across socioeconomic levels and geographic areas. Our research provides an approach to assessing health disparities by estimating the underlying respiratory infection regardless of symptomatic status. It may also provide a more accurate picture of viral infection in the community since many people are asymptomatic and many symptomatic people are unable or choose not to get tested.

The primary limitation of our study is that decedents are not a fully representative sample of the catchment population, as decedents are more likely to be male, non-Hispanic Black, and 18–64 years old compared to the catchment population, as discussed above [9]. Accordingly, it is possible that associations between the community characteristics of SVI and ADI and respiratory virus infection are different in the general population, and thus, a different public health strategy for community targeting would be appropriate. In future work, we plan to assess the extent to which the selection bias for decedents affects prevalence estimates and associations for the catchment population. Further, it is unknown whether our results are generalizable to other urban areas. Detroit and its surroundings have a troubled history of racial segregation (include redlining and “white flight”) [35], which might mean that some of the associations we found are particular to this area and are not fully generalizable to other urban areas. Similar work will need to be done in other locations.

We also did not have information about individual pre-existing health conditions, immunization or other prior immunity, smoking status, or other individual-level factors that may potentially influence the respiratory viral infection. Another limitation is that despite a wide range of social vulnerability and economic deprivation across the catchment area, decedents disproportionately came from more vulnerable and deprived communities, reducing our power to detect patterns across the full range of the SVI themes and ADI. Also, there is potential for misclassification bias as were unable to verify the extent to which the address provided to the MEO for each decedent was an accurate measure of their typical exposure; for example, a decedent may have lived in one location but worked in another. Finally, the BioFire RP2.1 panel used in this analysis is FDA-approved for the detection of viral nucleic acid from nasopharyngeal swabs of living people rather than decedents, and it is possible that the viral load for some decedents had decayed beyond the limit of detection, creating false negatives.

## Conclusion

We found that overall social vulnerability and socioeconomic deprivation were largely not associated with the prevalence of respiratory viruses other than RV in decedents in a large, urban medical examiner’s office, and so these community-level measures may not be effective metrics for distributing public health resources in a way that reduces infection disparity. However, a stronger association was seen with the social vulnerability theme of Minority Status & Language, and we recommend public health decision makers consider taking this metric into account when geographically distributing resources, such as limited vaccine supplies or community clinics. We did find that community-level social vulnerability and socioeconomic deprivation were associated with the prevalence of RV, and the reasons behind this association (and the lack of association for other respiratory viruses) should be examined in future work. Ultimately, our study builds a foundation to better understand disparities in respiratory virus infection, not just medically attended cases of disease, highlights the potential utility of infectious disease decedent surveillance for public health, and underscores the need to reduce disparities in infection prevalence across racial and ethnic, socioeconomic, and geographic strata.

## Supplementary Information

Below is the link to the electronic supplementary material.

Supplementary file1 (DOCX 37 KB)

## Data Availability

Data will be provided upon reasonable request to the corresponding author.

## Code Availability

Code will be provided upon reasonable request to the corresponding author.
