# Supplementary material for: Associations Between Community Social Vulnerability or Socioeconomic Deprivation and Respiratory Virus Infection in Decedents in a Large, Urban Medical Examiner’s Office
Source: J Racial Ethn Health Disparities. 2025 May 20;13(4):3077–87. doi: 10.1007/s40615-025-02482-x (PMC13346123; doi:10.1007/s40615-025-02482-x)
Supplement: Supplementary file 1 — Supplementary file1 (DOCX 37 KB) [file 40615_2025_2482_MOESM1_ESM.docx]

**Supplemental Table S1**. Sensitivity analysis of model fit and parsimony, as measured by the Bayesian Information Criterion (BIC), as a function of the degrees of freedom (DF) for the spline modeling the association between Social Vulnerability Index (SVI), SVI subthemes and ADI with infection with each of adenovirus, SARS-CoV-2, parainfluenza virus 2, rhinovirus, and respiratory syncytial virus prevalence. For every model, the using 3 degrees of freedom resulted in the lowest BIC.

| Virus and predictor | Degrees of freedom | | |
| --- | --- | --- | --- |
|  | 3 | 4 | 5 |
| **Adenovirus** |  |  |  |
| ADI | 1320.537 | 1327.227 | 1335.188 |
| SVI | 1329.852 | 1335.045 | 1342.599 |
| SVI: Socioeconomic Status | 1324.753 | 1331.077 | 1336.138 |
| SVI: Household Composition and Disability | 1330.540 | 1337.915 | 1345.162 |
| SVI: Minority Status and Language | 1322.300 | 1330.290 | 1338.358 |
| SVI: Housing Type & Transportation | 1331.094 | 1336.522 | 1343.105 |
| **SARS-CoV-2** |  |  |  |
| ADI | 3037.822 | 3042.349 | 3051.724 |
| SVI | 3055.099 | 3062.884 | 3069.010 |
| SVI: Socioeconomic Status | 3057.033 | 3065.152 | 3073.062 |
| SVI: Household Composition and Disability | 3055.337 | 3058.441 | 3064.589 |
| SVI: Minority Status and Language | 3052.476 | 3057.158 | 3064.223 |
| SVI: Housing Type & Transportation | 3057.221 | 3065.211 | 3073.079 |
| **Parainfluenza virus 2** |  |  |  |
| ADI | 487.5319 | 495.6493 | 501.6746 |
| SVI | 492.7163 | 500.8299 | 508.2455 |
| SVI: Socioeconomic Status | 489.8438 | 492.5520 | 500.6971 |
| SVI: Household Composition and Disability | 494.1287 | 501.6857 | 502.0857 |
| SVI: Minority Status and Language | 490.5263 | 497.8634 | 505.8454 |
| SVI: Housing Type & Transportation | 491.4915 | 497.5457 | 504.8439 |
| **Rhinovirus** |  |  |  |
| ADI | 2358.573 | 2366.012 | 2373.023 |
| SVI | 2382.281 | 2388.920 | 2394.574 |
| SVI: Socioeconomic Status | 2373.597 | 2381.251 | 2387.550 |
| SVI: Household Composition and Disability | 2391.312 | 2398.872 | 2406.825 |
| SVI: Minority Status and Language | 2375.889 | 2381.735 | 2386.309 |
| SVI: Housing Type & Transportation | 2398.883 | 2407.022 | 2413.778 |
| **Respiratory syncytial virus** |  |  |  |
| ADI | 476.3197 | 484.4537 | 492.2310 |
| SVI | 475.3240 | 477.3640 | 486.0149 |
| SVI: Socioeconomic Status | 474.7389 | 482.8570 | 490.6361 |
| SVI: Household Composition and Disability | 476.1987 | 483.3602 | 488.7161 |
| SVI: Minority Status and Language | 469.7563 | 477.7329 | 484.2199 |
| SVI: Housing Type & Transportation | 476.0219 | 482.9785 | 490.8241 |

**Supplemental Table S2**. Associations between age, sex, racial/ethnicity, manner of death, Social Vulnerability Index (SVI), SVI subthemes and area deprivation index (ADI) with infection with each of adenovirus, SARS-CoV-2, parainfluenza virus 2, rhinovirus, and respiratory syncytial virus prevalence among decedents in the Wayne County Medical Examiner’s Office (2020–22). The table summarizes the prevalence ratios (PR) and their 95% CIs estimated in univariable Poisson models with robust error variance and in multivariable models accounting for all demographic decedent characteristic and the SVI subtheme Minority Status and Language (MSL).

|  | Adenovirus | | SARS-CoV-2 | | Parainfluenza Virus 2 | | Rhinovirus | | Respiratory Syncytial Virus | |
| --- | --- | --- | --- | --- | --- | --- | --- | --- | --- | --- |
| Characteristic | Univariable | Multivariable | Univariable | Multivariable | Univariable | Multivariable | Univariable | Multivariable | Univariable | Multivariable |
|  | PR (95%CI) | PR (95%CI) | PR (95%CI) | PR (95%CI) | PR (95%CI) | PR (95%CI) | PR (95%CI) | PR (95%CI) | PR (95%CI) | PR (95%CI) |
| Age (years) |  |  |  |  |  |  |  |  |  |  |
| 0–4 | **3.02 (1.64, 5.55)** | **2.26 (1.20, 4.27)** | 1.24 (0.87, 1.78) | **1.47 (1.01, 2.14)** | **3.59 (1.19, 10.8)** | 2.58 (0.78, 8.48) | **7.97 (6.26, 10.2)** | **6.80 (5.17, 8.94)** | **4.72 (1.87, 11.9)** | **4.65 (1.78, 12.1)** |
| 5–17 | **7.64 (4.20, 13.9)** | **6.74 (3.50,12.9)** | 1.40 (0.81, 2.41) | 1.41 (0.81, 2.46) | **12.4 (4.58, 33.6)** | **9.80 (3.26, 29.4)** | **5.58 (3.58, 8.47)** | **5.61 (3.61, 8.71)** | 4.34 (1.00, 18.9) | **4.11 (1.01, 16.7)** |
| 18–39 | **2.54 (1.79, 3.59)** | **2.36 (1.66, 3.36)** | 1.08 (0.91, 1.28) | **1.19 (1.00, 1.42)** | **2.29 (1.16, 4.52)** | **2.13 (1.04, 4.36)** | **2.29 (1.82, 2.89)** | **2.17 (1.71, 2.75)** | 1.61 (0.81, 3.20) | 1.543 (0.73, 3.26) |
| 40–64 | 1.0 (ref) | 1.0 (ref) | 1.0 (ref) | 1.0 (ref) | 1.0 (ref) | 1.0 (ref) | 1.0 (ref) | 1.0 (ref) | 1.0 (ref) | 1.0 (ref) |
| 65+ | 0.74 (0.36, 1.49) | 0.76 (0.38, 1.53) | 0.85 (0.65, 1.11) | 0.82 (0.63, 1.07) | 0.0 (—) | 0.0 (—) | 0.51 (0.30, 0.88) | 0.54 (0.31, 0.92) | 0.26 (0.03, 1.93) | 0.27 (0.04, 2.02) |
| Sex |  |  |  |  |  |  |  |  |  |  |
| Female | **1.51 (1.11, 2.04)** | **1.43 (1.05, 1.96)** | 1.03 (0.88, 1.21) | 1.00 (0.84, 1.17) | 2.27 (1.25, 4.11) | 2.04 (1.10, 3.77) | **1.28 (1.05, 1.55)** | 1.10 (0.91, 1.34) | 1.70 (0.92, 3.13) | 1.52 (0.82, 2.80) |
| Male | 1.0 (ref) | 1.0 (ref) | 1.0 (ref) | 1.0 (ref) | 1.0 (ref) | 1.0 (ref) | 1.0 (ref) | 1.0 (ref) | 1.0 (ref) | 1.0 (ref) |
| Race/ethnicity |  |  |  |  |  |  |  |  |  |  |
| Black | **1.69 (1.21, 2.36)** | 1.32 (0.86, 2.04) | **1.24 (1.05, 1.46)** | 1.11 (0.90, 1.37) | **4.38 (1.84, 10.4)** | **3.96 (1.33,11.8)** | **1.68 (1.36, 2.09)** | **1.32 (1.00, 1.73)** | **2.79 (1.27, 6.10)** | 2.13 (0.71, 6.36) |
| Hispanic | 1.88 (0.82, 4.31) | 1.65 (0.69, 3.97) | **1.52 (1.01, 2.30)** | 1.49 (0.98, 2.26) | 2.56 (0.31, 21.5) | 2.55 (0.30, 21.7) | **2.19 (1.35, 3.56)** | **1.98 (1.17, 3.35)** | **5.76 (1.54, 21.6)** | **5.17 (1.20, 22.2)** |
| White | 1.0 (ref) | 1.0 (ref) | 1.0 (ref) | 1.0 (ref) | 1.0 (ref) | 1.0 (ref) | 1.0 (ref) | 1.0 (ref) | 1.0 (ref) | 1.0 (ref) |
| Other | 0.76 (0.18, 3.10) | 0.59 (0.14, 2.46) | 1.37 (0.85, 2.22) | 1.28 (0.79, 2.06) | **9.26 (2.33, 36.8)** | **7.59 (1.83, 31.5)** | 1.65 (0.90, 3.05) | 1.27 (0.69, 2.32) | 4.63 (0.98, 21.8) | 3.91 (0.77, 19.9) |
| Manner of death |  |  |  |  |  |  |  |  |  |  |
| Natural | 1.0 (ref) | 1.0 (ref) | 1.0 (ref) | 1.0 (ref) | 1.0 (ref) | 1.0 (ref) | 1.0 (ref) | 1.0 (ref) | 1.0 (ref) | 1.0 (ref) |
| Accident | **1.47 (1.02, 2.11)** | 1.28 (0.89, 1.84) | 0.64 (0.55, 0.76) | 0.61 (0.51, 0.72) | 1.31 (0.65, 2.62) | 1.23 (0.61, 2.48) | **1.87 (1.48, 2.38)** | **1.44 (1.13, 1.83)** | 1.21 (0.61, 2.39) | 0.98 (0.47, 2.06) |
| Violent | **2.40 (1.52, 3.80)** | 1.54 (0.95, 2.49) | 0.73 (0.55, 0.95) | 0.63 (0.48, 0.84) | 2.32 (0.97, 5.57) | 1.32 (0.50, 3.47) | **2.03 (1.46, 2.82)** | 1.27 (0.91, 1.78) | 1.62 (0.62, 4.19) | 1.06 (0.40, 2.80) |
| Indeterminate/ pending | **2.12 (1.16, 3.87)** | 1.57 (0.86, 2.89) | 0.66 (0.45, 0.97) | 0.57 (0.38, 0.85) | 1.10 (0.25, 4.89) | 0.68 (0.13, 3.40) | **2.68 (1.86, 3.86)** | 1.21 (0.85, 1.72) | 0.51 (0.07, 3.92) | 0.24 (0.03, 1.83) |
| SVI: overall |  |  |  |  |  |  |  |  |  |  |
| 0.0–0.20 | 1.0 (ref) | — | 1.0 (ref) | — | 1.0 (ref) | — | 1.0 (ref) | — | 0.00 (0.00, 0.00) | — |
| 0.21–0.40 | 0.95 (0.44, 2.04) | — | 1.20 (0.78, 1.86) | — | 0.74 (0.17, 3.31) | — | 1.12 (0.55, 2.26) | — | 1.0 (ref) | — |
| 0.41–0.60 | 0.61 (0.26, 1.45) | — | 1.25 (0.81, 1.93) | — | 0.82 (0.18, 3.64) | — | **2.34 (1.23, 4.45)** | — | 0.47 (0.12, 1.81) | — |
| 0.61–0.80 | 0.87 (0.44,1.73) | — | 1.25 (0.84, 1.84) | — | 0.47 (0.12, 1.88) | — | **1.96 (1.06, 3.60)** | — | 0.42 (0.15, 1.20) | — |
| 0.81–1.00 | 1.03 (0.54, 1.96) | — | 1.28 (0.88, 1.86) | — | 1.03 (0.31, 3.39) | — | **2.26 (1.25, 4.08)** | — | 0.73 (0.32, 1.68) | — |
| SVI: Socioeconomic Status |  |  |  |  |  |  |  |  |  |  |
| 0.0–0.20 | 1.0 (ref) | — | 1.0 (ref) | — | 1.0 (ref) | — | 1.0 (ref) | — | 1.0 (ref) | — |
| 0.21–0.40 | 0.59 (0.26, 1.30) | — | 1.26 (0.81, 1.96) | — | 1.09 (0.25, 4.86) | — | 0.82 (0.40, 1.69) | — | 0.20 (0.02, 1.84) | — |
| 0.41–0.60 | 0.37 (0.16, 0.84) | — | 1.02 (0.66, 1.58) | — | 0.76 (0.17, 3.40) | — | 1.15 (0.62, 2.13) | — | 0.43 (0.10, 1.91) | — |
| 0.61–0.80 | 0.60 (0.31, 1.15) | — | 1.28 (0.87, 1.89) | — | 0.37 (0.07, 1.81) | — | 1.65 (0.94, 2.88) | — | 0.64 (0.19, 2.18) | — |
| 0.81–1.00 | 0.77 (0.45, 1.33) | — | 1.22 (0.85, 1.76) | — | 1.00 (0.30, 3.26) | — | **1.88 (1.12, 3.17)** | — | 0.67 (0.23, 1.91) | — |
| SVI: household  composition & disability |  |  |  |  |  |  |  |  |  |  |
| 0.0–0.20 | 1.0 (ref) | — | 1.0 (ref) | — | 1.0 (ref) | — | 1.0 (ref) | — | 1.0 (ref) | — |
| 0.21–0.40 | 0.84 (0.43, 1.64) | — | 1.00 (0.69, 1.45) | — | 0.53 (0.15, 1.85) | — | 1.41 (0.88, 2.28) | — | 1.38 (0.41, 4.69) | — |
| 0.41–0.60 | 0.77 (0.42, 1.42) | — | 1.27 (0.92, 1.75) | — | 0.87 (0.32, 2.34) | — | 1.44 (0.93, 2.25) | — | 1.07 (0.33, 3.45) | — |
| 0.61–0.80 | 1.15 (0.67, 1.99) | — | 1.23 (0.90, 1.68) | — | 0.36 (0.12, 1.12) | — | **1.55 (1.01, 2.38)** | — | 0.72 (0.22, 2.39) | — |
| 0.81–1.00 | 0.90 (0.52, 1.56) | — | 1.18 (0.87, 1.60) | — | 0.75 (0.30, 1.91) | — | **1.55 (1.02, 2.36)** | — | 0.92 (0.30, 2.80) | — |
| SVI: minority status & language |  |  |  |  |  |  |  |  |  |  |
| 0.0–0.20 | 1.0 (ref) | 1.0 (ref) | 1.0 (ref) | 1.0 (ref) | 0.00 (0.00, 0,00) | 0.00 (0.00, 0.00) | 1.0 (ref) | 1.0 (ref) | 1.0 (ref) | 0.0 (0.0, 0.0) |
| 0.21–0.40 | 1.00 (0.24, 2.93) | 0.95 (0.34, 2.67) | 1.25 (0.65, 2.38) | 1.32 (0.69, 2.54) | 1.0 (ref) | 1.0 (ref) | 1.00 (0.34, 2.93) | 1.05 (0.36, 3.07) | 0.00 (0.00, 0.00) | 0.0 (0.0, 0.0) |
| 0.41–0.60 | 0.81 (0.30, 2.19) | 0.73 (0.27, 1.94) | 1.48 (0.82, 2.65) | 1.51 (0.83, 2.72) | 0.73 (0.12, 4.34) | 0.73 (0.12, 4.37) | 2.36 (0.95, 5.87) | 2.20 (0.89, 5.45) | 0.00 (0.00, 0.00) | 1.0 (ref) |
| 0.61–0.80 | 0.55 (0.20, 1.54) | 0.49 (0.18, 1.33) | 1.38 (0.77, 2.46) | 1.39 (0.77, 2.50) | 1.03 (0.20, 5.31) | 0.88 (0.19, 4.17) | 2.34 (0.95, 5.77) | 2.27 (0.92, 5.59) | 0.85 (0.25, 2.94) | 0.84 (0.24, 2.99) |
| 0.81–1.00 | 1.28 (0.53, 3.10) | 0.95 (0.39, 2.35) | 1.67 (0.97, 2.89) | 1.63 (0.92, 2.90) | 1.59 (0.38, 6.63) | 0.76 (0.19, 3.08) | **2.85 (1.19, 6.81)** | 2.20 (0.90, 5.34) | 1.24 (0.48, 3.17) | 0.82 (0.24, 2.77) |
| SVI: housing type and transportation |  |  |  |  |  |  |  |  |  |  |
| 0.0–0.20 | 1.0 (ref) | — | 1.0 (ref) | — | 1.0 (ref) | — | 1.0 (ref) | — | 1.0 (ref) | — |
| 0.21–0.40 | 0.98 (0.57, 1.68) | — | 1.02 (0.77, 1.36) | — | 0.75 (0.25, 2.21) | — | 1.16 (0.81, 1.65) | — | 1.31 (0.37, 4.62 | — |
| 0.41–0.60 | 0.84 (0.49, 1.42) | — | 1.13 (0.87, 1.47) | — | 0.69 (0.24, 1.97) | — | 1.17 (0.84, 1.64) | — | 1.73 (0.55, 5.51) | — |
| 0.61–0.80 | 1.16 (0.71, 1.89) | — | 1.05 (0.81, 1.36) | — | 0.83 (0.31, 2.23) | — | 1.11 (0.79, 1.55) | — | 1.62 (0.51, 5.15) | — |
| 0.81–1.00 | 0.96 (0.58, 1.59) | — | 1.12 (0.87, 1.45) | — | 1.21 (0.49, 2.99) | — | 1.17 (0.84, 1.62) | — | 1.67 (0.53, 5.23) | — |
| ADI |  |  |  |  |  |  |  |  |  |  |
| 0–20 | 0.66 (0.09, 4.87) | — | 0.40 (0.10, 1.60) | — | 0.00 (0.00, 0.00) | — | 0.52 (0.13, 2.08) | — | 2.66 (0.35, 20,2) | — |
| 21–40 | 0.57 (0.21, 1.54) | — | 1.00 (0.68, 1.47) | — | 1.08 (0.26, 4.51) | — | **0.51 (0.27, 0.97)** | — | 0.58 (0.08, 4.27) | — |
| 41–60 | 1.03 (0.63, 1.70) | — | 0.81(0.61, 1.08) | — | 1.15 (0.45, 2.94) | — | **0.38 (0.23, 0.63)** | — | 0.98 (0.35, 2.78) | — |
| 61–80 | 0.68 (0.43, 1.07) | — | 0.92 (0.75, 1.14) | — | 0.61 (0.24, 1.57) | — | **0.64 (0.48, 0.85)** | — | 0.78 (0.33, 1.88) | — |
| 81–100 | 1.0 (ref) | — | 1.0 (ref) | — | 1.0 (ref) | — |  |  | 1.0 (ref) | — |
